# FBG Monitoring Information-Motivated Anti-Fatigue Performance Analysis of CFRP Composites Based on Non-Destructive Tests

**DOI:** 10.3390/polym17131817

**Published:** 2025-06-29

**Authors:** Fu-Kang Shen, Si-Kai Wang, Jia-Yi Zhang, Zhi-Gang Xia, Bao-Rui Peng, Yung William Sasy Chan, Ping Xiang, Hua-Ping Wang

**Affiliations:** 1 School of Civil Engineering and Mechanics, Lanzhou University, Lanzhou 730000, China; shenfk19@lzu.edu.cn (F.-K.S.); wangsk19@lzu.edu.cn (S.-K.W.); xiazhg21@lzu.edu.cn (Z.-G.X.); 2 College of Architectural Science and Engineering, Yangzhou University, Yangzhou 225127, China; sasychanwill@yahoo.com; 3 School of Civil Engineering, Central South University, Changsha 410075, China; pxiang2-c@my.cityu.edu.hk; 4 Key Laboratory of Mechanics on Disaster and Environment in Western China, Ministry of Education, Lanzhou University, Lanzhou 730000, China

**Keywords:** CFRP plate, anti-fatigue performance, FBGs in series, fast assessment, online evaluation

## Abstract

The wide-spread application of carbon fiber-reinforced polymer (CFRP) composites in industrial fields has led to high demand for developing a rapid detection method for assessing the structural performance of CFRP composites in operation based on optical fiber sensing technology. Therefore, the effectiveness and reliability of evaluating the fatigue resistance of CFRP plates based on fiber Bragg grating (FBG) monitoring information were explored. The strain response of CFRP plates at key positions under constant amplitude fatigue load was monitored by bare FBGs in series and packaged quasi-distributed FBGs in series. The structural performance and fatigue resistance characteristics of CFRP plates were evaluated by statistical analysis and fatigue life prediction theory. The validity and accuracy of the test and analysis results were demonstrated by finite element modeling analysis. Compared with the traditional methods that evaluate the structural fatigue performance based on mass destructive experiments, this method significantly improves the detection efficiency and realizes the non-destructive and rapid online evaluation of structural service performance. Research shows that the designed FBG sensors can effectively monitor the strain response of CFRP plate under fatigue load, and the correlated fatigue algorithm can provide feasible and reliable technical approaches for online detection and evaluation on the structural performance of CFRP components.

## 1. Introduction

With the rapid development of modern industrial technology, lightweight, high-strength, and corrosion-resistant composite materials, particularly carbon fiber-reinforced polymers (CFRPs), have become essential materials in fields such as aerospace, rail transportation, automobile manufacturing, and bridge construction [1]. CFRP structures—with their advantages of low weight, high strength and stiffness, corrosion resistance, small thermal expansion coefficient, stability, excellent fatigue resistance, design flexibility (the ability to adjust material properties by altering components, contents, and fiber layout), and ease of overall molding—are widely used for structural reinforcement and the enhancement of service performance. However, during the service life of structures, many components are frequently subjected to repeated cyclic loading. Although the stress level of each load cycle may be significantly lower than the material’s yield strength, the cumulative effect of cyclic loading can gradually induce microstructural damage within the material, ultimately leading to fatigue failure. This form of failure is characterized by its latent nature, sudden onset, and strong dependence on the number of load cycles, making it one of the primary causes of long-term structural performance degradation and potential safety hazards [2,3]. Therefore, research on how to quickly and effectively detect the performance of CFRP plates is of great scientific and engineering significance [4,5,6].

Traditional methods for detecting CFRP plate performance are time-consuming and inefficient, making real-time detection and monitoring of in-service CFRP structures difficult [7,8,9,10,11,12,13,14,15,16,17]. For example, Wen et al. [13] explored the feasibility of using resistive measurement technology for continuous detection of CFRP damage states, but this method is highly susceptible to environmental temperature variations, leading to lower measurement accuracy. Munoz et al. [14] combined acoustic emission technology, which captures acoustic signals from damage events, with infrared thermography to detect thermal changes caused by damage in CFRP. However, both techniques generate large amounts of data, and the identification process is cumbersome, requiring complex pattern recognition and data processing methods. Li et al. [15] introduced a pulsed eddy current thermography method to detect damage in CFRP-reinforced steel structures. While this non-contact technology can quickly produce intuitive thermal imaging results, its equipment is expensive, and its data post-processing is complex. Additionally, pulsed eddy current thermography is sensitive to the electromagnetic properties of materials, particularly the fiber patterns of CFRP components, which can affect the accuracy of damage identification. Therefore, there is a need for further research to develop a rapid, efficient, and online detection method for CFRP structures.

In recent years, with the development of structural health monitoring (SHM) technology, fiber Bragg grating (FBG) sensors, due to their unique sensing advantages, have shown great potential in this field [18,19,20,21,22]. FBG sensors are small in size, lightweight, immune to electromagnetic interference, corrosion-resistant, intrinsically safe, easy to embed within composite materials, capable of multi-point and multi-parameter monitoring, and have good long-term stability, making them especially suitable for stress and strain monitoring in composite materials [23,24,25,26,27,28,29,30]. By embedding FBG sensors within a structure or attaching them to the surface, strain responses under fatigue loads can be monitored in real time, allowing for the assessment of fatigue performance. Wang et al. [25] proposed using discrete FBG sensor test data to reconstruct the real-time strain field of CFRP plates under static and dynamic loads, providing technical support for the visual representation of microdefects or damage. Li et al. [27] embedded FBG sensors in CFRP and recorded strain responses from the embedded sensors during hundreds of impact tests, improving the accuracy and reliability of impact damage identification through machine learning algorithms. Chen et al. [28] tested and analyzed the vibration response of CFRP plates with surface-mounted FBG sensors under various dynamic loads, investigating the dynamic response characteristics of CFRP structures and the sensing performance of FBG sensors through time-domain and frequency-domain analysis. These studies demonstrate the feasibility of using FBG sensors to detect the dynamic behavior of structures, showing that this technology can effectively and accurately assess the service performance of CFRP structures and play a positive role in promoting the design and development of smart composite material structures.

In view of this, a rapid detection method for structural performance of CFRP plates based on FBG monitoring information is developed. This method monitors the strain response of a CFRP plate under fatigue load in real time through an FBG sensor and combines statistical analysis and fatigue strength theory to diagnose and evaluate its fatigue strength and life prediction.

## 2. Rapid Detection Method for the Service Performance of CFRP Composites

To meet the demand for online rapid detection of the service performance of CFRP structures, a fast detection method based on optical fiber sensing technology is proposed for CFRP plate structures. FBG sensors can capture the real-time strain response of structures during the loading process. By comparing the strain data measured by the FBG sensors with the material’s ultimate strain and applying strength theory, the instantaneous strength of CFRP plates can be quickly estimated, enabling rapid detection of their service performance. The advantage of this method is that it can provide a structural performance evaluation in a short time, without the need for time-consuming destructive testing. Additionally, by integrating long-term monitoring strain data analysis with fatigue strength theory algorithms, the anti-fatigue performance of the CFRP plate structure can be assessed.

### 2.1. Experimental Description

The CFRP material used in this experiment was a standard industrial-grade carbon fiber-reinforced epoxy resin composite with a specific fiber volume fraction and porosity. The dimensions of the CFRP plate were 500 mm × 100 mm × 2 mm, with a material elastic modulus of 150 GPa, Poisson’s ratio of 0.3, and density of 1.55 g/cm^3^. The layout sequence of the multiple CFRP strips was [0/90/0/90/0/90]. The two ends of the CFRP plate were fixed using clamps, forming a simply supported constraint at both ends. To evaluate the anti-fatigue performance of the CFRP plate, FBG sensors were attached to specified locations on the plate surface to measure the response under cyclic loading. To check the sensing performance of the proposed FBG sensors bonded on the CFRP plate, fatigue tests with different loading cycles were performed in sequence. Therefore, a case study in Stage 1 with short loading cycles was initially conducted to check and validate the possibility of the proposed method. Based on the fundamental analysis, case studies in Stage 2 and Stage 3 were further implemented.

**Figure 1:**
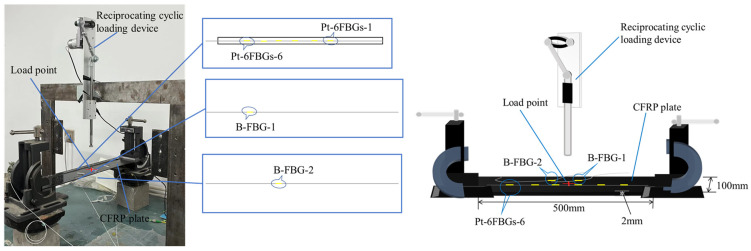
FBG sensor layout during the test.

Considering that packaged FBG sensors are commonly used in practical engineering tests, two types of FBG sensors were employed in this experiment: (1) a silicone rubber packaged FBG array containing six measurement points, labeled Pt-6FBGs (FBGs in series 1–6), and (2) a bare FBG array with two measurement points, labeled B-FBG-1 and B-FBG-2. The locations of the FBG sensors are shown schematically in Figure 1. Flexible silicone rubber was used to attach the FBG sensors to ensure that the deformation transmission path between the sensors and the CFRP plate was flawless and that the deformation occurred in unison.

**Figure 2:**
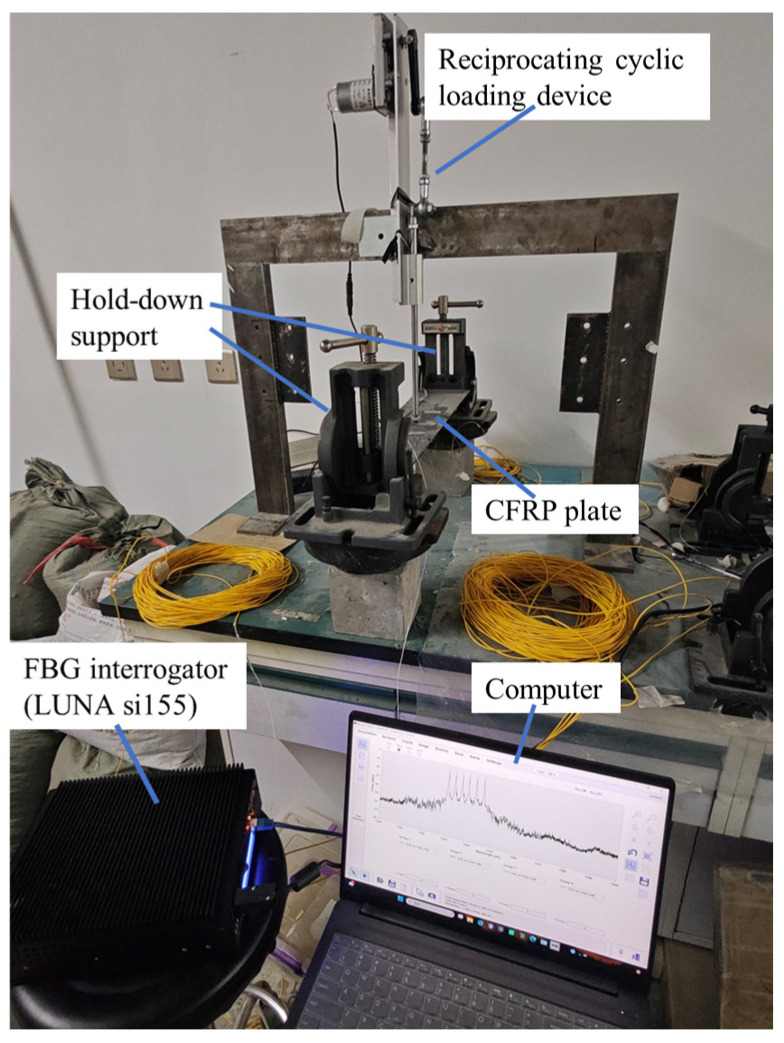
Data collection process.

A simple cyclic loading device was used to apply fatigue loading to the CFRP plate. The loading point was located at the center of the plate surface, with constant amplitude loading at a frequency of 0.75 Hz. The first loading test lasted for 100 h, while the second repeated test lasted 138 h. Prior to testing, the FBG sensors were connected to an optical fiber interrogator with the sampling frequency set to 1000 Hz. Initial data were collected for more than 5 min to record the baseline wavelength before the cyclic loading began. The experimental setup and data acquisition system are shown in Figure 2.

### 2.2. Data Processing of FBG Sensing Signals

The wavelength data collected by the optical fiber demodulator are processed based on the relationship between the wavelength shift and strain to obtain the strain values at the corresponding positions of the CFRP plate [30]. The time is converted into load cycles for analysis. The relationship between strain and wavelength shift is given by

(1) $$\varepsilon =\frac{1}{\lambda_{B}k_{\varepsilon }}\left(\Delta \lambda_{B}-\frac{\Delta \lambda_{\mathrm{BT}}\beta }{\alpha +\beta }\right)$$

where ε is the strain at the corresponding position of the CFRP plate; $\lambda_{B}$ is the center wavelength of the FBG; $k_{\varepsilon }$ is the strain sensitivity coefficient; $\Delta \lambda_{B}$ is the wavelength shift, which is the difference between the current wavelength and the initial wavelength; $\Delta \lambda_{\mathrm{BT}}$ is the wavelength shift caused by temperature variation; α is the thermal expansion coefficient of the fiber; β is the thermo-optic coefficient. Since all the experimental studies were conducted in a laboratory with stable room temperature, the temperature effect on the FBG sensing elements is ignored, which means that $\Delta \lambda_{\mathrm{BT}}$ is 0.

Using the obtained strain, Hooke’s law is applied to calculate the structural stress at different strain levels. Then, combining strength theory, the instantaneous strength of the structure is estimated and compared with the material’s ultimate strength to diagnose the physical state of the structure.

### 2.3. Monitoring Data Analysis

#### 2.3.1. Strain Response of the CFRP Plate Under Fatigue Loading in One Cycle

**Figure 3:**
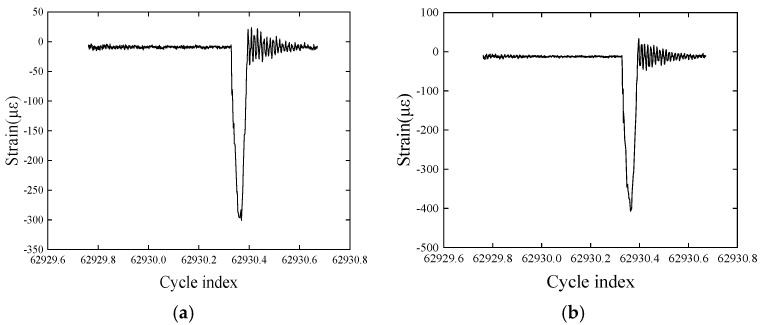

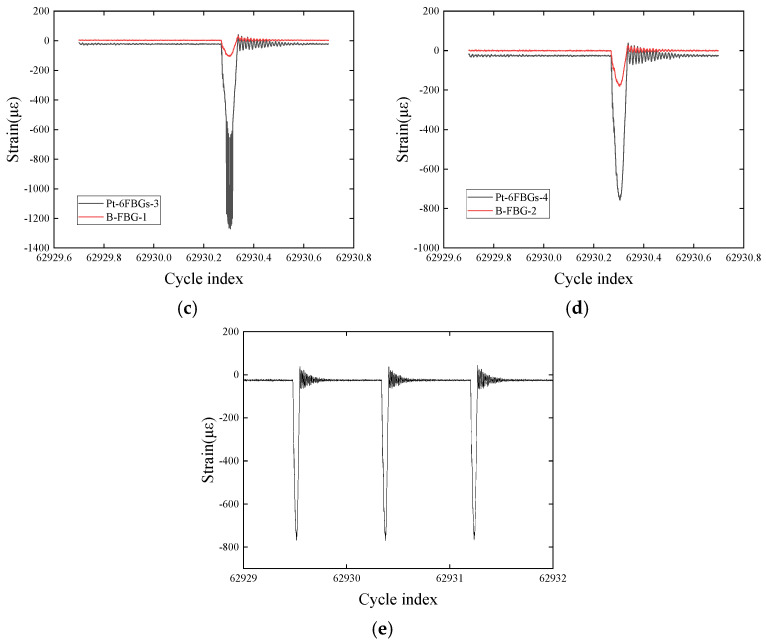
Strain diagram measured by different FBG sensors: (**a**–**d**) one-cycle period; (**e**) multiple-cycle period. (**a**) Pt-6FBGs-2, (**b**) Pt-6FBGs-5, (**c**) Pt-6FBGs-3 and B-FBG-1, (**d**) Pt-6FBGs-4 and B-FBG-2, (**e**) Pt-6FBGs-4.

After processing the wavelength data, the strain change trend within one cycle is observed at six measurement points, Pt-6FBGs-2, Pt-6FBGs-3, Pt-6FBGs-4, Pt-6FBGs-5, B-FBG-1, and B-FBG-2, as shown in Figure 3a–d. It can be observed that in the initial stage, when the loading rod is not in contact with the CFRP plate, the strain at the measurement points remains stable with almost no change. When the loading rod contacts the plate and moves downward, the measurement points on the plate’s surface experience compression, reflected as negative strain values, which gradually increase with the movement of the loading rod. When the loading rod reaches the lowest point, the maximum strain value is observed. As the loading rod moves upward, the strain value gradually decreases, showing symmetry in the loading and unloading processes. When the loading rod detaches from the CFRP plate, due to the rebound effect of the plate, the strain at the measurement points inside the plate shows significant fluctuations, alternating between positive and negative, indicating that the measurement points are subjected to cyclic vibrations. Afterward, the vibrations gradually weaken, and the strain stabilizes, returning to the initial value, entering the next loading cycle.

The strain–cycle number curves at the FBG measurement points clearly reflect the dynamic response characteristics of the CFRP plate during the cyclic loading process. The results show that the FBG sensors can accurately capture the strain variations at any given moment, a feature that enables FBG sensors to monitor the structural status of CFRP plates in real time. This allows for rapid and effective detection of the structural performance of CFRP plates under different working conditions. In any given cycle, the maximum strain value of the CFRP plate is analyzed, revealing that this strain is much lower than the ultimate compressive strain of the CFRP plate. This result indicates that the CFRP plate has not yet reached the failure strength state, thus validating the effectiveness and reliability of the rapid detection method for the service performance of CFRP plate structures. Using this detection method, the strength condition of the CFRP plate can be quickly determined within a short time, providing scientific support for the structural health assessment.

#### 2.3.2. Strain Response of the CFRP Plate in the Whole Fatigue Loading Process

**Figure 4:**
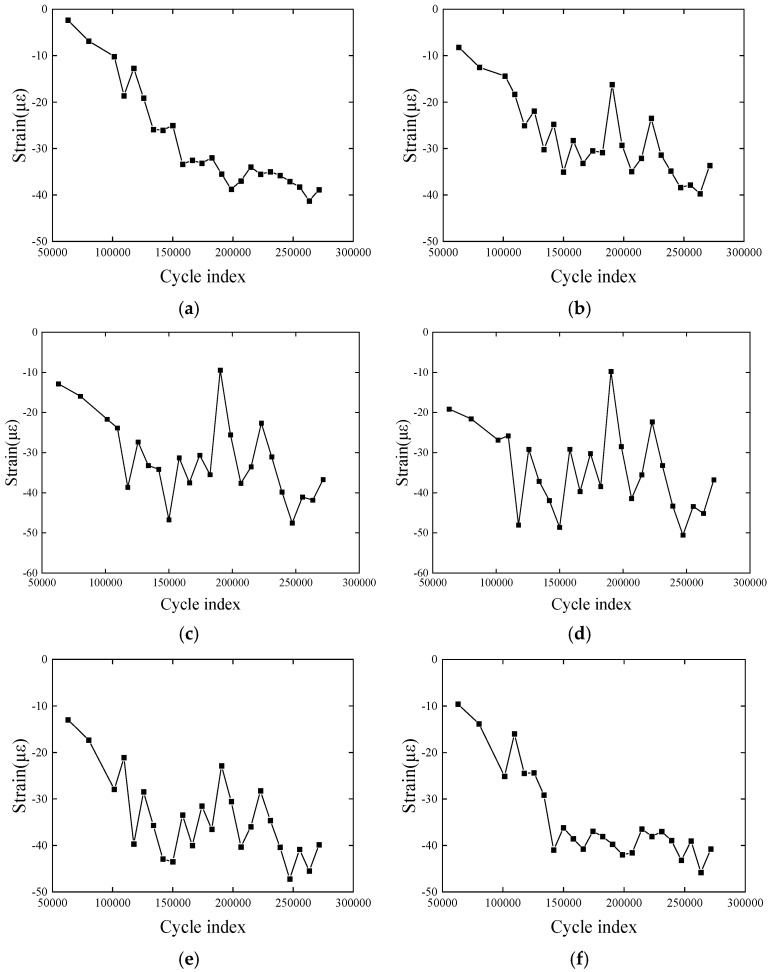

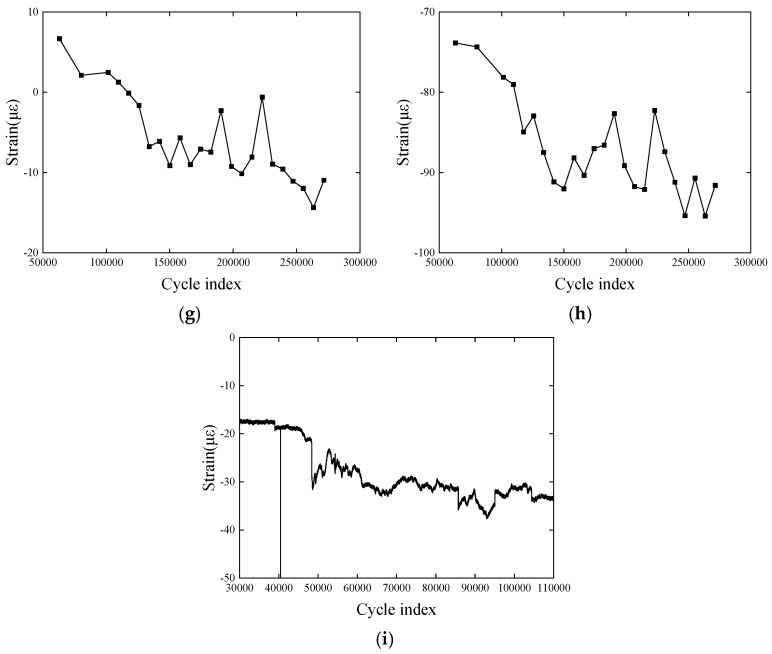
Strain distribution in the first fatigue loading process: (**a**–**h**) variation in average strains; (**i**) variation in original strains. (**a**) Pt-6FBGs-1, (**b**) Pt-6FBGs-2, (**c**) Pt-6FBGs-3, (**d**) Pt-6FBGs-4, (**e**) Pt-6FBGs-5, (**f**) Pt-6FBGs-6, (**g**) B-FBG-1, (**h**) B-FBG-2, (**i**) Pt-6FBGs.

Figure 4 shows the strain response of the CFRP plate throughout the entire experimental process, with strain increasing as the number of cycles increases. This reflects the microdefect effects that accumulate in the material under repeated loading, and the strain fluctuates slightly, which is closely related to the material’s vibration rebound behavior. Since the measured values are micro-strains, the value reaches 25 με only after 100,000 cycles, which is within a reasonable range. The strain growth rate changes slowly as the cycle number increases, indicating that cyclic hardening and softening phenomena have occurred inside the composite material. These are common material behaviors during fatigue processes. The non-linear strain growth characteristics and the potential hardening and softening effects highlight the complex response of the material under fatigue loading. This experimental result demonstrates that FBG sensors can accurately capture minute structural changes. Moreover, it also proves the feasibility and reliability of the proposed rapid detection method throughout the entire fatigue loading process.

**Figure 5:**
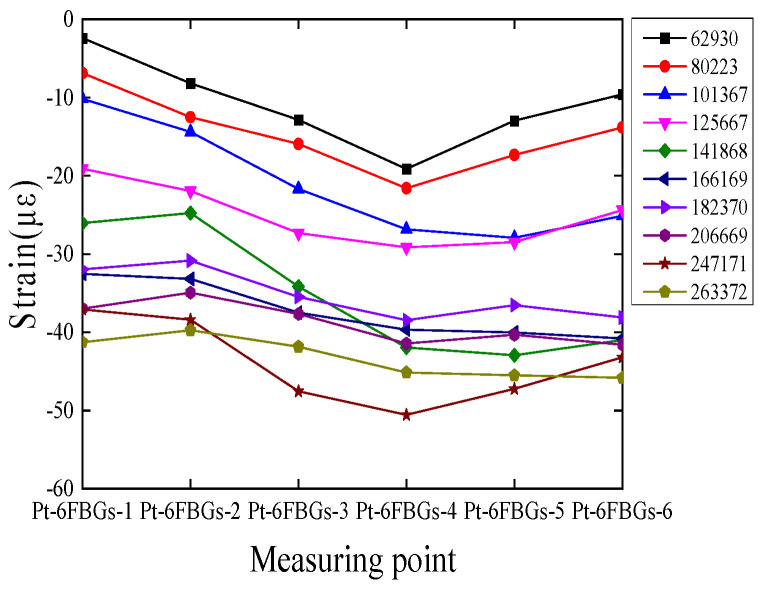
Strain distributions of the packaged FBGs in series at different loading cycle indexes in the first fatigue test.

Figure 5 shows the strain response of the CFRP plate measured by the packaged optical fiber sensor, displaying the distribution along the plate’s length at different loading cycle indexes. Based on the longitudinal arrangement of the packaged optical fiber sensor along the CFRP plate, the strain data at discrete points intuitively reflect the morphological changes in the plate during the test. In the initial stage, the strain variation in the CFRP plate is symmetrically distributed with respect to the neutral axis of the plate, showing a good symmetric distribution. The strain values gradually decrease from the center of the plate to the sides, with a clear strain gradient. As the cyclic loading increases, the previously symmetrical strain curve gradually becomes asymmetric, and a decrease in strain values appears at local measurement points. This indicates that microstructural changes occur inside the plate under long-term loading, leading to a redistribution of strain. Overall, the behavior is consistent with the expected mechanical behavior of CFRP structures.

**Figure 6:**
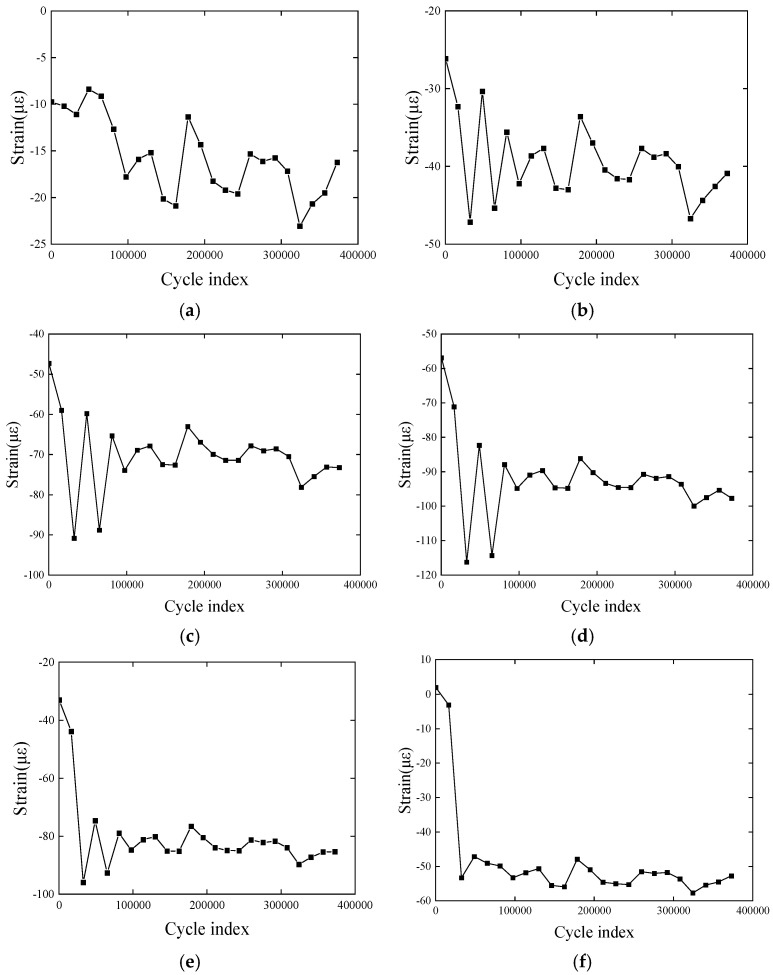

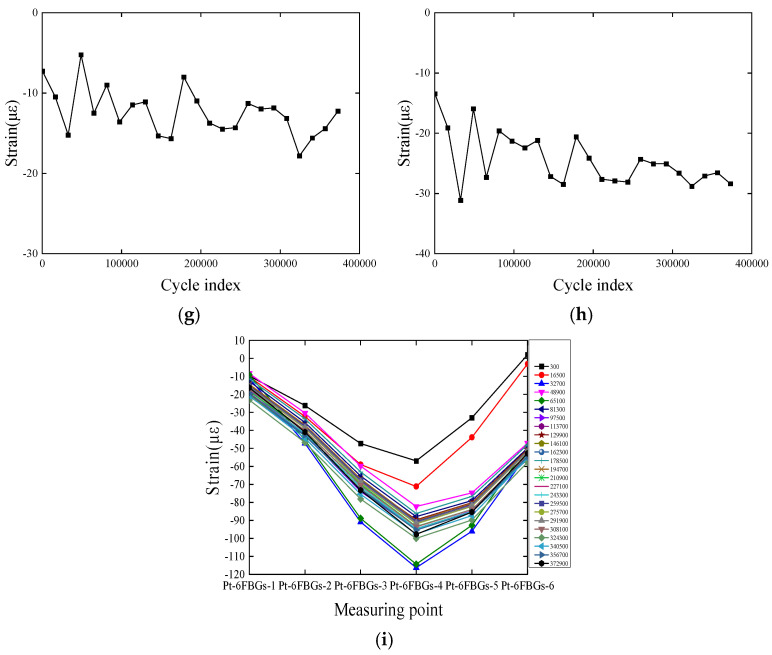
Strain distribution in the second fatigue loading process: (**a**–**h**) strain–cycle diagram in the whole test process; (**i**) strain distribution of the packaged FBGs in series at different loading cycle indexes. (**a**) Pt-6FBGs-1, (**b**) Pt-6FBGs-2, (**c**) Pt-6FBGs-3, (**d**) Pt-6FBGs-4, (**e**) Pt-6FBGs-5, (**f**) Pt-6FBGs-6, (**g**) B-FBG-1, (**h**) B-FBG-2.

By comparing Figure 6 with the strain distribution from the first experiment, we find that in the second repeated fatigue loading test, the strain values at the same locations on the plate are higher than those from the first test. The strain values consistently increase over time, indicating that the sensors can continuously and accurately capture the actual strain changes of the structure. The successful execution of the repeated test and the consistent strain distribution trends in both tests (i.e., the strain values at the measured locations on the CFRP plate increase with the number of cycles) demonstrate the feasibility of FBG sensors in long-term monitoring. Both the packaged FBG sensors and the bare FBG sensors exhibit good continuous sensing performance, making them suitable for long-term strain monitoring.

#### 2.3.3. Strain Response Characteristics Between the Packaged and Bare FBG Sensors

**Figure 7:**
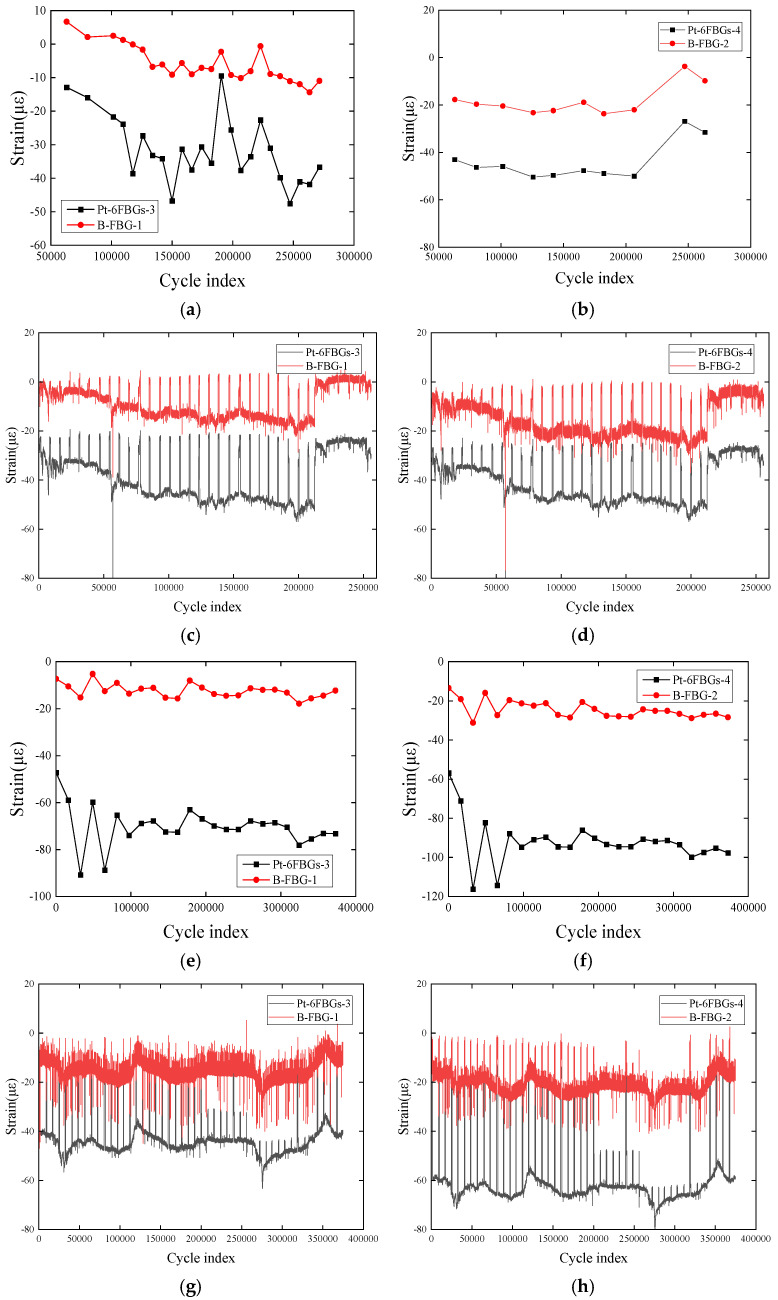
Comparison of strain trends between packaged and bare FBG sensors: (**a**–**d**) first fatigue test; (**e**–**h**) second fatigue test. (**a**) Comparison of strain trends between Pt-6FBGs-3 and B-FBG-1, (**b**) comparison of strain trends between Pt-6FBGs-4 and B-FBG-2, (**c**) original strain changes in Pt-6FBGs-3 and B-FBG-1, (**d**) original strain changes in Pt-6FBGs-4 and B-FBG-2, (**e**) comparison of strain trends between Pt-6FBGs-3 and B-FBG-1, (**f**) comparison of strain trends between Pt-6FBGs-4 and B-FBG-2, (**g**) original strain changes in Pt-6FBGs-3 and B-FBG-1, (**h**) original strain changes in Pt-6FBGs-4 and B-FBG-2.

Figure 7 shows a comparative analysis of the strain response monitoring of the CFRP plate by using the packaged FBG sensor Pt-6FBGs and the bare optical fiber sensor B-FBG. The analysis results show that both types of sensors exhibit good consistency in monitoring the strain response of the CFRP plate, with slight differences in the measured strain values due to the influence of the packaging layer. The packaged optical fiber sensor may slightly reduce sensitivity but improves the stability and reliability of the measurement. Due to its protective layer, the packaged optical fiber sensor is suitable for long-term strain monitoring in harsh environments. In summary, both packaged FBG sensors and bare FBG sensors demonstrate good continuous sensing performance. The sudden strain increases in Figure 7c,d may be attributed to the external disturbance. Since the sharp changes in strain disappear quickly, it can be concluded that no practical effect occurs in the sensor or the CFRP plate. In actual measurement processes, the selection of the appropriate health monitoring sensor type for CFRP plates or other composite material structures should consider the characteristics of the structure being tested as well as environmental factors.

## 3. Verification Analysis Based on Finite Element Simulation

This paper constructs a finite element model of the CFRP plate to simulate the corresponding fatigue loading tests. By comparing the finite element simulation results with experimental data, the effectiveness and reliability of the FBG sensor monitoring data are verified.

### 3.1. Finite Element Modeling Analysis

To facilitate the numerical simulation, the following assumptions are adopted: the CFRP plate is modeled as an orthotropic linear elastic material, and geometrical and material non-linearities are neglected. Ideal fixed supports have been applied at both ends, and quasi-static loading is assumed. Environmental effects such as temperature and humidity are ignored due to the stable testing environment in laboratory. For fatigue life prediction, a stress-based fatigue analysis (S–N method) is employed by using standardized material data, without accounting for damage accumulation, stiffness degradation, or crack propagation.

**Figure 8:**
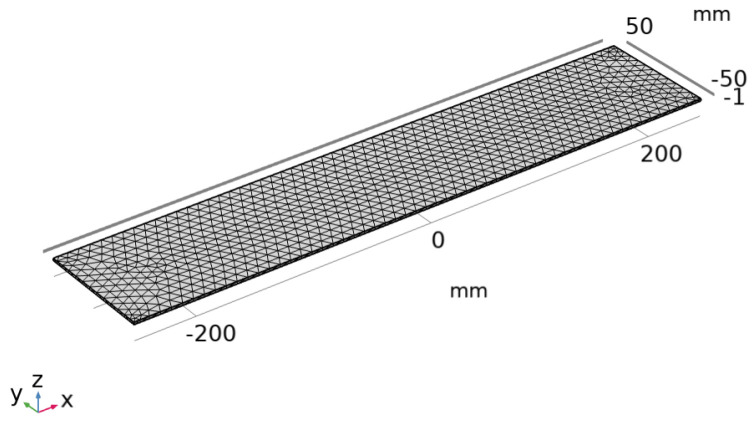
Establishment of CFRP plate model.

On this basis, the analysis model was constructed by using COMSOL Multiphysics 6.1 finite element software (as shown in Figure 8). The modeling process includes the following steps:

(1) Create the geometry model: construct an accurate geometry model based on the actual dimensions and shape of the CFRP plate.
(2) Define material properties: set the material’s elastic modulus and Poisson’s ratio and apply boundary conditions with fixed ends.
(3) Mesh generation: generate a mesh for the geometry model, ensuring sufficient refinement in key areas while avoiding overly dense meshes that may lead to excessive computational load.
(4) Set monitoring points: locate the positions of the FBG sensors within the model and set monitoring points at these locations to collect strain data.
(5) Configure the solver: choose appropriate numerical methods and solver types, conduct transient analysis, and set reasonable solution steps and time steps.
(6) Post-processing analysis: visualize parameters such as strain, stress, and damage, and configure output formats for comparison with experimental data.

**Figure 9:**
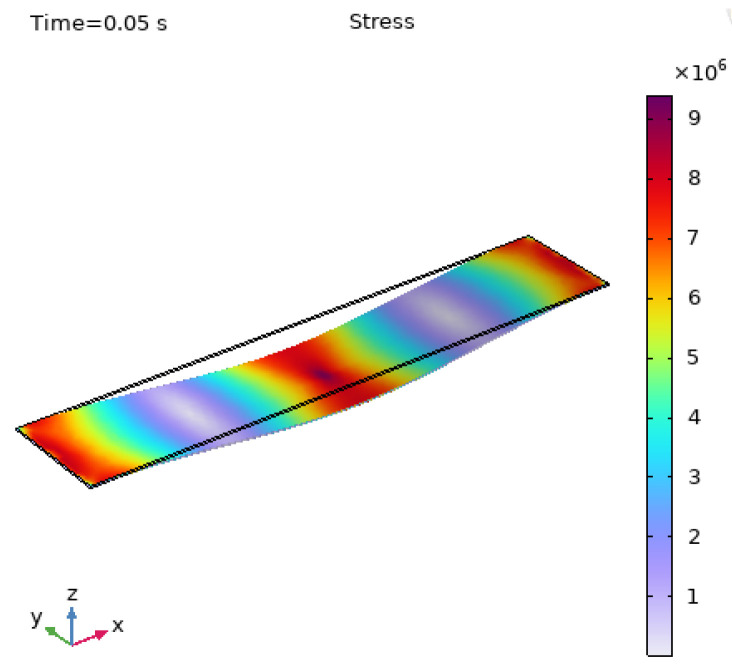
Stress distribution based on finite element simulation analysis.

In COMSOL, a stress analysis of the CFRP plate under the experimental conditions is simulated to evaluate the stress and strain distribution of the structure under fatigue loading. A transient study is first conducted, as shown in Figure 9, with the finite element analysis results at t = 0.05 s (the moment when the cyclic loading device is fully applied to the plate), which helps analyze the material’s response characteristics under dynamic loading conditions. Figure 9 shows the stress distribution characteristics of the CFRP plate under specific loading conditions. The uneven and concentrated stress phenomena provide important information for understanding the material’s mechanical behavior and predicting its performance in practical applications.

### 3.2. Comparative Analysis

**Figure 10:**
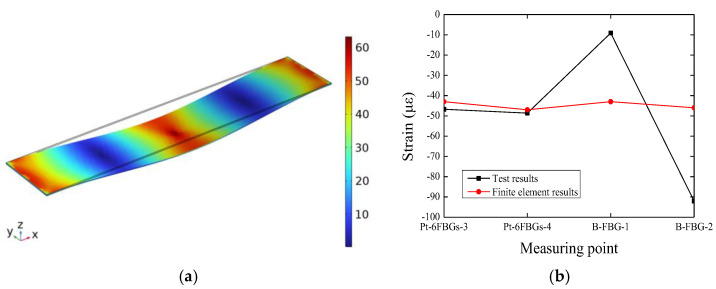
Analysis result and comparison diagrams corresponding to the measuring points. (**a**) Diagram of strains. (**b**) Strain profiles.

Discussion on the finite element analysis at the four measurement points—Pt-6FBGs-3, Pt-6FBGs-4, B-FBG-1, and B-FBG-2—is performed. As shown in Figure 10, the simulated strain at the measurement point of Pt-6FBGs-3 under specific conditions is 43 με, and the simulated strain at the Pt-6FBGs-4 measurement point is 47 με. These values align well with the experimental data, further validating the effectiveness and reliability of the FBG monitoring data. In contrast, the simulated strains at the B-FBG-1 and B-FBG-2 measurement points are 43 με and 46 με, respectively. The differences to the experimental data may be related to environmental factors (such as temperature, humidity, or mechanical interference), which could significantly affect sensing performance in the absence of protective layer. It indicates that improvement and optimization can be adopted to enhance the monitoring quality of the proposed FBG sensors.

The consistency between simulated and experimental data indicates that the packaged optical fiber sensor has higher measurement reliability under harsh environmental conditions, supporting its applicability for long-term strain monitoring and providing important feedback for the ongoing improvement of sensing technology.

**Figure 11:**
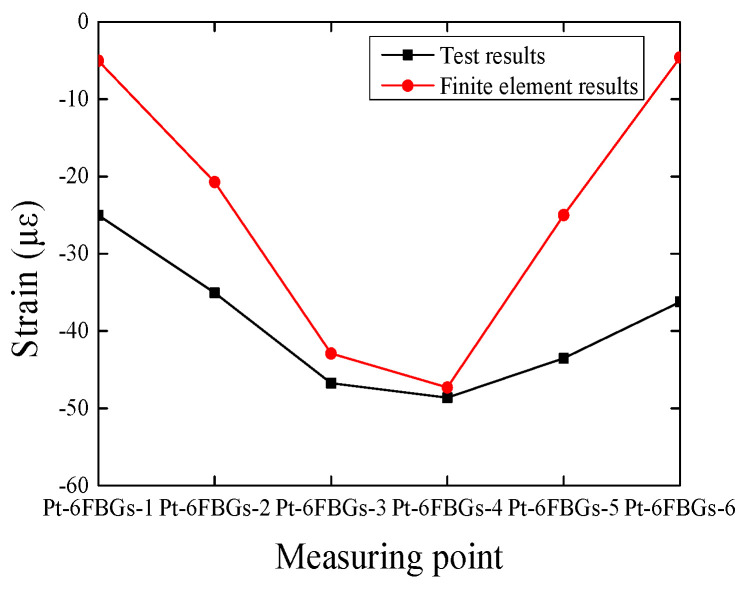
Comparison diagram between the results measured by packaged optical fiber sensor and the finite element results.

Figure 11 shows the comparison between the experimental response data of the six packaged optical fiber sensors (Pt-6FBGs-1 to Pt-6FBGs-6) and the finite element analysis results. It can be observed that the finite element analysis results align well with the experimental data from the packaged optical fiber sensors, further validating the reliability and stability of the packaged optical fiber sensors in monitoring the strain of the CFRP plate.

## 4. Fatigue Resistance Evaluation of CFRP Structures Based on FBG Monitoring Information

The above contents prove the feasibility of the FBG sensing monitoring method in the rapid detection of CFRP plate performance and the effectiveness and reliability of FBG monitoring data. Based on the fatigue calculation theory algorithm, the long-term monitoring data were analyzed to evaluate the fatigue resistance of CFRP plate structure, and the results were compared with the fatigue resistance obtained by finite element analysis to verify the feasibility of evaluating the fatigue resistance of CFRP plates based on FBG sensing information. The practical loading mode for a composite component may be random with no loading sequence effect. The stress level of the composite components can be obviously lower than the yield value and the microcomposites may be damaged in the elastic deformation stage under the dynamic actions, which weakens the non-linear behavior of the composite component. Therefore, Miner’s linear damage accumulation theory was adopted for analysis at this stage. The concept is different from the traditional concept on exploring the fatigue properties of composites based on destructive tests, which intends to establish the online anti-fatigue performance assessment system based on monitoring data of composite structures in service.

### 4.1. Fatigue Strength

Fatigue strength is an index that characterizes the ability of materials to resist fatigue failure under cyclic loading. It is usually expressed as the maximum stress amplitude or strain amplitude that the material can withstand under specific loading conditions (such as stress ratio, number of cycles, etc.).

In this study, the fatigue life standard is $N_{f}$ = 10^6^ (typical fatigue life standard), and the corresponding fatigue stress amplitude is calculated by Basquin’s law [31,32]. The Basquin’s law formula is

(2) $$\Delta \sigma =\sigma_{f}^{\prime }{\left(2N_{f}\right)}^{b}$$

where Δσ is the stress amplitude, $N_{f}$ is the fatigue life, $\sigma_{f}^{\prime }$ is the fatigue strength coefficient, and b is the fatigue strength index. In this study, $\sigma_{f}^{\prime }$ is 1500 MPa, b is −0.1, and the result is Δσ = 377 MPa. Therefore, the fatigue strength of the CFRP plate material in this study is 377 MPa under $N_{f}$ = 10^6^.

### 4.2. Fatigue Life Prediction Based on Monitoring Information

Based on the existing experimental data, the change trend of strain with the number of cycles shows the damage accumulation process of CFRP plate under fatigue load. In order to further evaluate the fatigue resistance of the material, this study uses the Miner rule (linear fatigue damage accumulation theory) to predict the fatigue life. The Miner rule [33,34,35] is a classical fatigue life prediction model that evaluates the fatigue life of materials by estimating the damage accumulation of materials under cyclic loading. The Miner rule assumes that the damage of the material accumulates linearly during the fatigue loading process. When the damage accumulation reaches a critical value (usually set to 1), the material will fail. The basic formula is

(3) $$D=\underset{i}{\sum }\frac{n_{i}}{N_{f,i}}$$

where D is the damage accumulation (D = 1 at failure), $n_{i}$ is the number of loading cycles under a certain strain amplitude, and $N_{f,i}$ is the fatigue life under this strain amplitude. According to Miner’s rule, the cumulative damage $D_{i}$ of each stage is calculated.

According to the experimental data (the strain change trend in Figure 4 and Figure 6), the fatigue process can be divided into three stages (taking Figure 4 as an example):

(1) Stage 1 (initial stage, 0–50,000 times): strain change is small, no significant fatigue damage;
(2) Stage 2 (damage acceleration stage, 50,000–150,000 times): the strain amplitude increased significantly, indicating that the material stiffness decreased;
(3) Stage 3 (steady state stage, 150,000–270,000 times): the strain growth rate slows down, and the strain amplitude tends to be stable.

Hooke’s law is applied to transform the strain amplitude into the stress amplitude. The basic formula is

(4) Δσ=E⋅Δε

where *E* is the elastic modulus of the material, and Δε is the strain amplitude. Formula (3) is further used to predict the fatigue life $N_{f,i}$ of each stage.

According to the Miner’s rule, when the damage accumulation is D = 1, the material will experience fatigue failure. Assuming that the damage accumulation rate remains unchanged at each stage, total fatigue life of the material $N_{f}$ can be calculated. Table 1 and Table 2 show the fatigue life prediction results based on the experimental data and Miner’s rule.

Taking the minimum fatigue life, it can be obtained that the fatigue life of the CFRP plate $N_{f}$ = 2.05 × 10^20^ under the condition of this study.

### 4.3. Fatigue Life Prediction Based on Simulation Analysis

**Figure 12:**
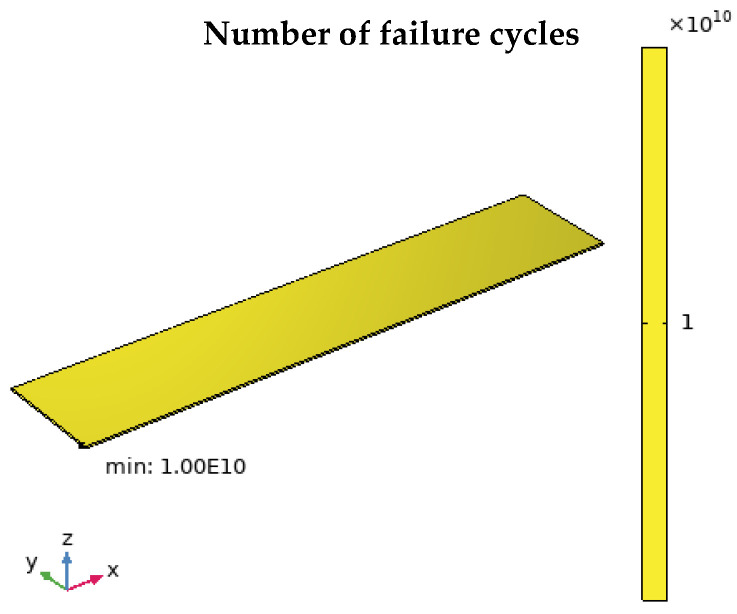
Fatigue life prediction in finite element analysis.

The fatigue life prediction of the CFRP plate under this test condition is carried out by using finite element analysis. Based on the S-N curve of CFRP plates [7], combined with the stress distribution obtained by simulation calculation, the fatigue life is predicted. The finite element analysis results are shown in Figure 12. Under the action of 10 N load, the CFRP plate can withstand up to 10^10^ cyclic loads, showing that it has significant fatigue life and excellent fatigue resistance.

The fatigue life predicted by the finite element analysis is 10^10^ times, which is significantly lower than the 2.05 × 10^20^ times predicted by the experimental analysis. This difference is mainly due to the calculation limitation of the finite element model, that is, it cannot predict more than 10^10^ times the fatigue life. However, based on the analysis, it is difficult for the material to fail under this fatigue load. Therefore, although the fatigue life prediction of finite element analysis is low, the trend of the two is consistent, which indicates that the CFRP plate has excellent fatigue resistance, which also verifies the feasibility of evaluating the fatigue resistance of CFRP plates based on FBG sensing information.

## 5. Conclusions

This study successfully developed a rapid and online detection method for structural performance of CFRP plates based on FBG monitoring information and evaluated the fatigue resistance of CFRP plates based on non-destructive tests. The following conclusions were drawn from this study:

(1) Fatigue resistance evaluation of a CFRP plate based on long-term monitoring information was carried out. By combining FBG monitoring data and Miner’s rule, the fatigue life of the CFRP plate under a fatigue load with constant amplitude is predicted to be 2.05 × 10^20^ times by experimental analysis. According to the experimental results, the fatigue strength of CFRP plate material under *N_f_* = 10^6^ cycles is 377 MPa. Although the prediction value of fatigue life based on the finite element analysis is significantly lower than the experimental prediction result, both of them show that the CFRP plate has excellent fatigue resistance. These results verify the feasibility of evaluating the fatigue resistance of CFRP plates based on FBG sensing information. Future research should focus on improving the fatigue life prediction model and optimizing the accuracy of material parameters in the model to improve the prediction accuracy.

(2) The effectiveness and long-term stability of the proposed FBG sensors has been proved for monitoring health condition of CFRP composites under fatigue loads. Based on the strain response of a single measuring point, the stress–strain behavior of the CFRP plate during the loading and unloading process can be described in detail, including compression, rebound effect, and strain fluctuation. The strain response of the FBGs in series arranged longitudinally along the CFRP plate shows the expected strain symmetry and gradient in material mechanics, which verifies that the FBG sensor has excellent online monitoring ability. These results show that the FBG sensor can not only measure and explain the dynamic response of the CFRP plate in real time to evaluate the service state of the structure in real time but also can quickly and effectively detect the service performance of the structure under different working conditions. Through the successful implementation of the repeated test and the comparative analysis with the initial test, the stability of the FBG sensor in long-term monitoring is further verified. Both the encapsulated FBG sensor and the bare FBG sensor show excellent continuous sensing performance and are suitable for long-term strain monitoring.

(3) The strain measurement results of the packaged FBG sensor are highly consistent with the finite element analysis results, which verifies its effectiveness in fatigue performance monitoring of CFRP plates. In contrast, due to the lack of protection, the mechanical strain of the bare FBG sensor during the installation process and the change in ambient temperature during the test may lead to a deviation between the measured data and the actual test value. This phenomenon reveals the potential impact of environmental factors on measurement accuracy. It is generally suggested that the packaged FBG sensors can be adopted in the fatigue tests to obtain much stable and accurate information Therefore, it is necessary to further improve and optimize the monitoring method of FBG sensors to improve measurement accuracy and long-term reliability.

Based on the monitoring data of FBG sensors, this study successfully realized the rapid detection of structural performance in CFRP plates. Combined with statistical analysis and fatigue strength theory, this study effectively evaluated the fatigue strength of CFRP plates and predicted their fatigue life. This method provides a new technical approach for the online health monitoring and fatigue life assessment of composite structures.

## Figures and Tables

**Table 1:** Fatigue life prediction based on the monitoring information of the first fatigue test.

Measurement Point	Stage	Δε	Δσ	$N_{f,i}$	$D_{i}$	$\Sigma D_{i}$	$N_{f}$
**Pt-6FBGs-1**	Stage 1 (0–50,000)	2 × 10^−6^	3 × 10^5^	9.77 × 10^36^	5.12 × 10^−33^	4.03 × 10^−20^	6.69 × 10^24^
	Stage 2 (50,000–160,000)	20 × 10^−6^	3 × 10^6^	9.77 × 10^26^	1.13 × 10^−22^		
	Stage 3 (160,000–270,000)	36 × 10^−6^	5.4 × 10^6^	2.74 × 10^24^	5.13 × 10^−21^		
**Pt-6FBGs-2**	Stage 1 (0–50,000)	8 × 10^−6^	1.2 × 10^6^	9.31 × 10^30^	5.37 × 10^−27^	1.38 × 10^−20^	1.96 × 10^25^
	Stage 2 (50,000–150,000)	22 × 10^−6^	3.3 × 10^6^	3.77 × 10^26^	2.66 × 10^−22^		
	Stage 3 (150,000–270,000)	32 × 10^−6^	4.8 × 10^6^	8.88 × 10^24^	1.35 × 10^−20^		
**Pt-6FBGs-3**	Stage 1 (0–50,000)	13 × 10^−6^	1.95 × 10^6^	7.25 × 10^28^	6.89 × 10^−25^	2.9 × 10^−20^	9.32 × 10^24^
	Stage 2 (50,000–150,000)	29 × 10^−6^	4.35 × 10^6^	2.38 × 10^25^	4.21 × 10^−21^		
	Stage 3 (150,000–270,000)	34 × 10^−6^	5.1 × 10^6^	4.84 × 10^24^	2.48 × 10^−20^		
**Pt-6FBGs-4**	Stage 1 (0–50,000)	19 × 10^−6^	2.85 × 10^6^	1.63 × 10^27^	3.07 × 10^−23^	5.92 × 10^−20^	4.56 × 10^24^
	Stage 2 (50,000–150,000)	33 × 10^−6^	4.95 × 10^6^	6.53 × 10^24^	1.53 × 10^−20^		
	Stage 3 (150,000–270,000)	36 × 10^−6^	5.4 × 10^6^	2.74 × 10^24^	4.39 × 10^−20^		
**Pt-6FBGs-5**	Stage 1 (0–50,000)	13 × 10^−6^	1.95 × 10^6^	7.25 × 10^28^	6.89 × 10^−25^	6.59 × 10^−20^	4.1 × 10^24^
	Stage 2 (50,000–150,000)	31 × 10^−6^	4.65 × 10^6^	1.22 × 10^25^	8.2 × 10^−21^		
	Stage 3 (150,000–270,000)	37 × 10^−6^	5.55 × 10^6^	2.08 × 10^24^	5.77 × 10^−20^		
**Pt-6FBGs-6**	Stage 1 (0–50,000)	10 × 10^−6^	1.5 × 10^6^	1 × 10^30^	5 × 10^−26^	1.37 × 10^−19^	1.97 × 10^24^
	Stage 2 (50,000–140,000)	25 × 10^−6^	3.75 × 10^6^	1.05 × 10^26^	8.58 × 10^−22^		
	Stage 3 (140,000–270,000)	40 × 10^−6^	6 × 10^6^	9.54 × 10^23^	1.36 × 10^−19^		
**B-FBG-1**	Stage 1 (0–50,000)	7 × 10^−6^	1.05 × 10^6^	3.54 × 10^31^	1.41 × 10^−27^	4.34 × 10^−26^	6.23 × 10^30^
	Stage 2 (50,000–150,000)	5 × 10^−6^	7.5 × 10^5^	1.02 × 10^33^	9.77 × 10^−29^		
	Stage 3 (150,000–270,000)	9 × 10^−6^	1.35 × 10^6^	2.87 × 10^30^	4.18 × 10^−26^		
**B-FBG-2**	Stage 1 (0–50,000)	74 × 10^−6^	1.11 × 10^7^	2.03 × 10^21^	2.46 × 10^−17^	6.18 × 10^−16^	4.37 × 10^20^
	Stage 2 (50,000–150,000)	84 × 10^−6^	1.26 × 10^7^	5.72 × 10^20^	1.75 × 10^−16^		
	Stage 3 (150,000–270,000)	90 × 10^−6^	1.35 × 10^7^	2.87 × 10^20^	4.18 × 10^−16^		

**Table 2:** Fatigue life prediction based on the monitoring information of the second fatigue test.

Measurement Point	Stage	Δε	Δσ	$N_{f,i}$	$D_{i}$	$\Sigma D_{i}$	$N_{f}$
**Pt-6FBGs-1**	Stage 1 (0–70,000)	10 × 10^−6^	1.5 × 10^6^	1 × 10^30^	7 × 10^−26^	9.68 × 10^−23^	3.93 × 10^27^
	Stage 2 (70,000–160,000)	17 × 10^−6^	2.55 × 10^6^	4.96 × 10^27^	1.81 × 10^−23^		
	Stage 3 (160,000–380,000)	18 × 10^−6^	2.7 × 10^6^	2.8 × 10^27^	7.86 × 10^−23^		
**Pt-6FBGs-2**	Stage 2 (0–100,000)	37 × 10^−6^	5.55 × 10^6^	2.08 × 10^24^	4.81 × 10^−20^	4.24 × 10^−19^	8.96 × 10^23^
	Stage 3 (100,000–380,000)	41 × 10^−6^	6.15 × 10^6^	7.45 × 10^23^	3.76 × 10^−19^		
**Pt-6FBGs-3**	Stage 2 (0–100,000)	70 × 10^−6^	1.05 × 10^7^	3.54 × 10^21^	2.82 × 10^−17^	1.19 × 10^−16^	3.18 × 10^21^
	Stage 3 (100,000–380,000)	71 × 10^−6^	1.07 × 10^7^	3.07 × 10^21^	9.11 × 10^−17^		
**Pt-6FBGs-4**	Stage 2 (0–100,000)	90 × 10^−6^	1.35 × 10^7^	2.87 × 10^20^	3.49 × 10^−16^	1.86 × 10^−15^	2.05 × 10^20^
	Stage 3 (100,000–380,000)	94 × 10^−6^	1.41 × 10^7^	1.86 × 10^20^	1.51 × 10^−15^		
**Pt-6FBGs-5**	Stage 2 (0–100,000)	72 × 10^−6^	1.08 × 10^7^	2.67 × 10^21^	3.74 × 10^−17^	5.27 × 10^−16^	7.21 × 10^20^
	Stage 3 (100,000–380,000)	84 × 10^−6^	1.26 × 10^7^	5.72 × 10^20^	4.9 × 10^−16^		
**Pt-6FBGs-6**	Stage 2 (0–40,000)	25 × 10^−6^	3.75 × 10^6^	1.05 × 10^26^	3.81 × 10^−22^	5.95 × 10^−18^	6.39 × 10^22^
	Stage 3 (40,000–380,000)	53 × 10^−6^	7.95 × 10^6^	5.72 × 10^22^	5.95 × 10^−18^		
**B-FBG-1**	Stage 2 (0–160,000)	12 × 10^−6^	1.8 × 10^6^	1.62 × 10^29^	9.91 × 10^−25^	7.35 × 10^−24^	5.17 × 10^28^
	Stage 3 (160,000–380,000)	14 × 10^−6^	2.1 × 10^6^	3.46 × 10^28^	6.36 × 10^−24^		
**B-FBG-2**	Stage 2 (0–160,000)	23 × 10^−6^	3.45 × 10^6^	2.41 × 10^26^	6.63 × 10^−22^	5.19 × 10^−21^	7.32 × 10^25^
	Stage 3 (160,000–380,000)	37 × 10^−6^	4.05 × 10^6^	4.86 × 10^25^	4.53 × 10^−21^		

## Data Availability

The data supporting the results reported in the paper can be accessed from the corresponding authors.
